# Sucrose dampens caffeine-induced blood pressure elevations – A randomized crossover pilot study in healthy, non-obese men

**DOI:** 10.3389/fnut.2022.896055

**Published:** 2022-08-04

**Authors:** Yann Ravussin, Jean-Pierre Montani, Erik Konrad Grasser

**Affiliations:** Department of Endocrinology, Metabolism, and Cardiovascular System, University of Fribourg, Fribourg, Switzerland

**Keywords:** caffeine, cardiovascular, sucrose, hemodynamic, blood pressure, soft drinks, cardiac effects, glucose metabolism

## Abstract

**Purpose:**

Sales for sugar-sweetened and caffeinated beverages are still rising globally and their consumption has been linked to the development of cardiovascular diseases. However, direct evidence from human interventional studies in response to such beverages is still scarce.

**Methods:**

Seven young, non-obese men participated in a randomized crossover study where four test drinks [60 g sucrose + 50 mg caffeine, 60 g sucrose + caffeine-placebo, 50 mg caffeine, and caffeine-placebo] were investigated. Each drink was brought to a total volume of 500 mL with water. Continuous and beat-to-beat hemodynamic monitoring was conducted for 30 min baseline and continued for 90 min after the ingestion of each drink. Measurements included blood pressure, heart rate, stroke volume, cardiac output, total peripheral resistance, index of contractility, and double product.

**Results:**

Two-factor ANOVA analysis revealed significant treatment-by-time effects for diastolic blood pressure, heart rate, stroke volume, cardiac output, total peripheral resistance, index of contractility, and double product (all *p* < 0.01). Diastolic blood pressure and total peripheral resistance increased significantly to caffeine-only (all *p* < 0.05), while sucrose + caffeine-placebo and sucrose + caffeine both decreased resistance responses (all *p* < 0.05). Cardiac output increased significantly to sucrose + caffeine-placebo and sucrose + caffeine (all *p* < 0.05), and on trend for heart rate, stroke volume, and index of contractility (all *p* between 0.05 and 0.09).

**Conclusion:**

In young, non-obese men, a caffeinated and sucrose-sweetened beverage at concentrations similar to classical commercial Cola products exhibited distinct hemodynamic actions where the presence of sucrose dampened caffeine-induced blood pressure elevations, but at the expense of a tendency to increase cardiac work.

## Introduction

Sugar-sweetened beverages are still one of the most common sources of added sugars to diets in Western societies (1) as well as in middle- and low-income countries (2). Scientific evidence with respect to epidemiological and cross-sectional follow-up studies is accumulating that daily consumption of sugar-sweetened beverages is strongly associated with negative health outcomes. A recent cross-sectional investigation brought strong support for the notion that sugar-sweetened beverage consumption increases the risk of cardiovascular disease-related mortality in a dose-dependent manner (3). Not surprisingly, consumption of sugar-sweetened beverages is associated with the development of type 2 diabetes (4), which on its own acts as a driving force toward the development of cardiovascular diseases (5). Additionally, acute cardiovascular responses of energy drinks, i.e., highly caffeinated beverages with or without sugar, have been linked to an adverse hemodynamic profile with an augmented cardiac workload (6).

However, human studies looking at the synergistic effect of added sugars and caffeine are still scarce. Our laboratory, by using continuous and beat-to-beat hemodynamic monitoring, which has been suggested as a method of choice for similar human experiments (6), brought evidence that the caffeine and the sugar moiety contained in a commercially available energy drink affect the cardiovascular system through different pathways on myocardial and vascular properties (7). Moreover, our laboratory recently observed that the moiety of sugar (i.e., glucose, fructose, and sucrose) exhibits distinct hemodynamic effects where the glucose moiety contained in sucrose dampens the blood pressure elevating effects of fructose (8). This might be due to the actions of insulin in response to elevated blood glucose levels to oral glucose ingestion, which was observed to substantially lower vascular resistance in young and healthy men (9).

However, research is lacking about hemodynamic responses to a low-caffeinated and sucrose-sweetened beverage at a composition similar to commercially available beverages. We hypothesized that the addition of sucrose to a low-caffeinated beverage would modify caffeine-induced elevations in blood pressure. Therefore, a placebo-controlled, randomized crossover study was conducted in healthy, non-obese men to ascertain hemodynamic effects and potential interactions between the known cardiovascular dietary effectors sucrose and caffeine.

## Materials and methods

### Subjects

Seven young and non-obese men aged 25.6 ± 2.4 years were included in the study following recruitment from advertisements around the University’s campus and surrounding areas. Daily caffeine intake was estimated from a questionnaire and caffeine naïve individuals as well as heavy coffee drinkers (more than four cups a day) or regular energy drinks consumers were excluded from the study. A total of eight healthy non-obese men were initially included following a power analysis based on our previous study (7) that used the same experimental design (four treatments in a randomized crossover design, men only). Unfortunately, one subject dropped out of the study due to personal reasons and only seven subjects who completed all four arms of the study are presented here. The mean height of the subjects was 178 ± 6 cm, body weight was 78 ± 8 kg, and body mass index was 24.9 ± 1.9 kg ⋅ m^–2^. None of the subjects had any diseases or were taking any medication affecting cardiovascular regulation and subjects were explicitly advised to fast for at least 12 h and to abstain from alcohol, smoking, and caffeine as well as from vigorous exercise for 24 h prior to each test and were asked to maintain their normal dietary habits between the tests. This study was conducted according to the guidelines laid down in the Declaration of Helsinki, and all procedures involving human subjects were approved by the Commission Cantonale D’Ethique, Canton de Vaud, Switzerland, ethics number: 239/15. Written informed consent was obtained from all subjects, and all experiments were conducted in the human physiology ward at the University of Fribourg.

### Experimental design

All studies started between 08:00 and 08:30 in an air-conditioned (∼22°C) and quiet laboratory with subjects at thermal comfort. Every test subject attended four separate experimental sessions (separated by at least 3 days, but less than one week) according to a randomized crossover study design where the session’s order was determined before the study started^1^ (8). At each experimental session, hemodynamic responses to one of four drinks were monitored. Drinks tested were as follows: (*A*) water containing 60 g sucrose plus a caffeine placebo capsule, (*B*) water containing a 50 mg caffeine capsule, (*C*) water containing 60 g sucrose plus a 50 mg caffeine capsule, and (*D*) water containing a caffeine placebo capsule. Each drink was made up to a total of 500 mL by the addition of room temperature tap water. The content of sucrose and caffeine was chosen based on current concentrations (10, 11) found in worldwide market leaders for classical sugar-sweetened Cola products (i.e., Coca-Cola Inc. and PepsiCo Inc.) (12). The subjects were blinded regarding the caffeine ingestion, as the placebo capsule and the caffeine capsule were identical in appearance. The treatment (drink) sequence was known to the investigator but was not communicated to the subjects.

Upon arrival at the laboratory, subjects were asked to empty their bladders if necessary and sit in a comfortable armchair. The equipment was then attached and the test subject’s upper part of the body was covered with a light blanket (8). After waiting for a period of ∼30 min to attain stable values for cardiovascular functions, hemodynamic recordings started with a 30-min baseline after which each subject ingested the test drink over a period of 4 min, followed by post-drink cardiovascular recordings for a subsequent 90 min (8). Throughout the procedures, subjects watched non-stressful documentaries (8).

### Cardiovascular recordings

Continuous cardiovascular recordings were performed by using a Task Force Monitor (CNSystems, Medizintechnik, Graz, Austria) with a 1.000 Hz data sampling rate (8). Blood pressure was measured with the Penaz principle from either the index or middle finger of the right hand that was calibrated to oscillometric brachial blood pressure measurements on the contralateral arm (8). Both arms rested on ductile pillows that were placed on height adjustable tables with the reference of the fourth intercostal space to avoid drifts due to hydrostatic pressure gradients. Heart rate was measured by using a high-resolution six-channel electrocardiogram from which RR-intervals were continuously recorded. Stroke volume was assessed by impedance cardiography measurements performed based on the original Kubicek approach (13) but using an improved estimate of thoracic volume (14). Electrode strips were positioned in accordance with the manufacturer’s guidelines (CNSystems standard electrode kits). Myocardial contractility was derived through impedance cardiography measurements, at which the index of contractility reflects the aortic peak flow and the maximum impedance changes (ΔZ/Δt_*max*_) were normalized to ground impedance Z_0_ (8, 15).

### Data and statistical analysis

Beat-to-beat values of systolic blood pressure, diastolic blood pressure, heart rate, stroke volume, cardiac output, total peripheral resistance, index of contractility, and double product were averaged over the last 15 min of the baseline period and over every 15 min throughout the 90 min post drink period. Cardiac output was derived as the product of stroke volume and heart rate, and total peripheral resistance was calculated as mean arterial pressure/cardiac output. Double product was calculated as heart rate x systolic blood pressure and has been suggested as a marker for myocardial oxygen demand (16, 17).

All values are reported as means ± standard deviation for the seven participants. Statistical analysis was performed by two-way ANOVA for repeated measures with time (baseline, 15, 30, 45, 60, 75, and 90 min) and treatment [(*A*) sucrose-only (60 g); (*B*) sucrose (60 g) plus caffeine (50 mg); (*C*) caffeine-only (50 mg); (*D*) caffeine placebo control] as within-subject factors using statistical software (*GraphPrism* Version 7, St. Paul, United States). Where significant differences were observed, the effects of each drink over time were analyzed by comparing averaged data from every 15 min post-drink interval with the baseline data recorded during the 15 min immediately before drinking by using one-way repeated measures ANOVA with Holm-Sidak’s *post hoc* testing. The same statistical test was used to compare responses (i.e., averaged data over 90 min post-drink and subtracted by its baseline data) between the four drink types as well as to its baseline, respectively. Level of significance was set at *p* ≤ 0.05.

## Results

Baseline hemodynamic variables for the seven participants that concluded the experiments in 2015 and 2016 are presented in Table 1 and were not found to be significantly different between four treatments (all variables *p* > 0.05). One dropped out for unknown personal reasons. No adverse effects were observed during any test [i.e., no cardiac arrhythmia or tachycardia (i.e., heart rate > 100 beats ⋅ min^–1^) were noticed nor were any gastrointestinal symptoms reported].

**TABLE 1:** Continuously and beat-to-beat recorded baseline hemodynamic data averaged over the last 15 min of a 30 min baseline in seven healthy, non-obese men included in the study after recruitment who ingested four different drinks in a randomized crossover fashion.

	Sucrose + Placebo	Caffeine	Sucrose + Caffeine	Placebo
Systolic blood pressure, mmHg	123 ± 12	118 ± 10	120 ± 13	120 ± 12
Diastolic blood pressure, mmHg	76 ± 6	75 ± 7	75 ± 6	75 ± 7
Heart rate, beats ⋅ min^–1^	64 ± 8	63 ± 7	64 ± 9	63 ± 8
Double product, beats ⋅ min^–1^ ⋅mmHg	7862 ± 1273	7539 ± 1529	7705 ± 1741	7515 ± 1452
Stroke volume, mL	96 ± 15	92 ± 13	96 ± 19	97 ± 18
Index of contractility, 1000 ⋅ s^–1^	56 ± 8	53 ± 7	55 ± 10	56 ± 9
Cardiac output, L ⋅ min^–1^	6.1 ± 0.8	5.7 ± 0.5	6.1 ± 0.9	6.0 ± 0.8
Total peripheral resistance, mmHg ⋅ L^–1^⋅ min	15.2 ± 2.6	15.6 ± 2.3	15.0 ± 3.1	15.3 ± 3.0

No significant differences between baseline hemodynamic data were observed (all p > 0.10). Data are presented as means ± standard deviation.

### Directly measured hemodynamic parameters

**FIGURE 1:**
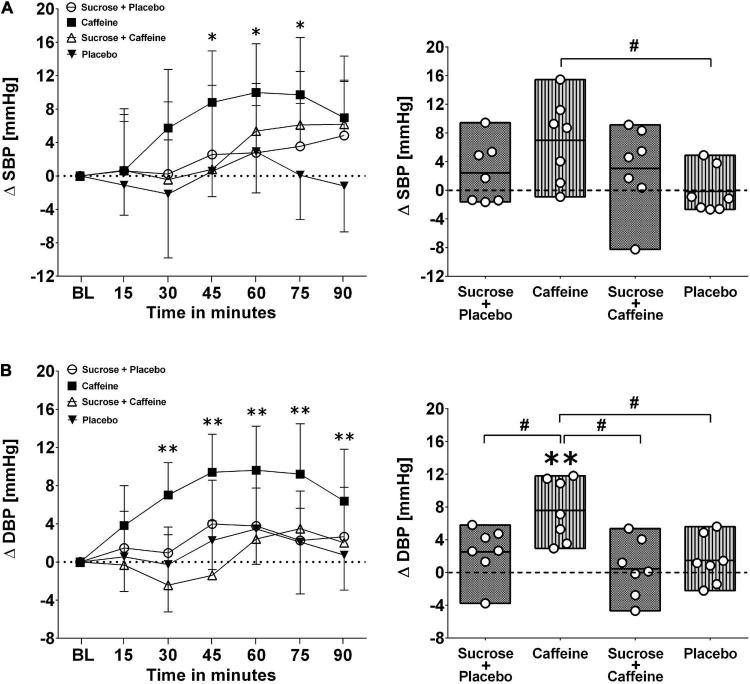
Left panels **(A,B)**: Time course for changes in systolic blood pressure (stocktickerSBP) and diastolic blood pressure (DBP) in response to the four drinks. Each mean value (i.e., 15-min blocks) corresponds to the average over the respective interval that was subtracted by its baseline (BL) values. Data are presented as means ± standard deviation. Right panels **(A,B)**: Boxes (minimum to maximum values) represent individual mean responses (open circles) averaged over 90 min post-drink and subtracted by baseline values for each drink, respectively. In both panels, asterisks show statistically significant differences over time from baseline values (both panels) at *p < 0.05 and **p < 0.01. For right panels only, **^#^***p* < 0.05 represents statistically significant differences between responses to the drinks as analyzed by one-way repeated measures analysis of variance with Holm–Sidak’s *post hoc* testing.

**FIGURE 2:**
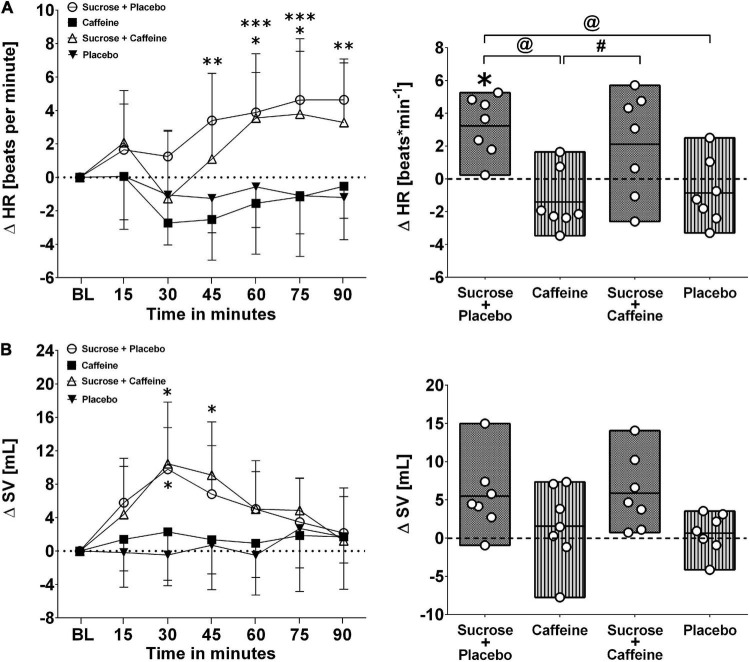
Left panels **(A,B)**: Time course for changes in heart rate (HR) and stroke volume (SV) in response to the four drinks. Each mean value (i.e., 15-min blocks) corresponds to the average over the respective interval that was subtracted by its baseline (BL) values. Data are presented as means ± standard deviation. Right panels **(A,B)**: Boxes (minimum to maximum values) represent individual mean responses (open circles) averaged over 90 min post-drink and subtracted by baseline values for each drink, respectively. In both panels, asterisks show statistically significant differences over time from baseline values (both panels) at *p < 0.05, **p < 0.01, and ***p < 0.005. For right panels only, **^#^***p* < 0.05 and **^@^***p* < 0.01 represent statistically significant differences between responses to the drinks as analyzed by one-way repeated measures analysis of variance with Holm–Sidak’s *post hoc* testing.

Figures 1, 2 present changes in systolic and diastolic blood pressure as well as changes in heart rate and stroke volume in response to the ingestion of four test drinks. Two-way repeated-measures ANOVA analysis provided evidence for a significant treatment versus time interaction effect in average responses to diastolic blood pressure (*p* < 0.005), heart rate (*p* < 0.005), and stroke volume (*p* < 0.005), while systolic pressure presented a trend (*p* = 0.06).

Ingestion of the caffeine-only drink increased significantly diastolic blood pressure starting from min 20 to 30 after the ingestion, peaking from min 45 to 60 (+ 9.6 ± 4.6 mmHg, *p* < 0.01), and gradually returning thereafter back to baseline values, a pattern that was not seen for other drinks. Moreover, diastolic blood pressure responses (i.e., over 90 min post-drink) revealed that the caffeine-only drink had significantly higher values compared to sucrose + caffeine (*p* = 0.01), sucrose + caffeine-placebo (*p* = 0.04), and the water + caffeine placebo negative control (*p* = 0.04). A similar pattern was observed for systolic blood pressure responses to caffeine-only and to a lesser magnitude for sucrose-only and sucrose + caffeine (Figure 1, panels A and B). On the other hand, sucrose-only (*p* < 0.005) and to lesser extent sucrose + caffeine (*p* < 0.05) increased integrated heart rate responses compared to caffeine-only. A similar pattern, however, not significantly different between the drink types, was seen for integrated stroke volume responses (Figure 2, panels A and B).

### Indirectly measured hemodynamic parameters

**FIGURE 3:**
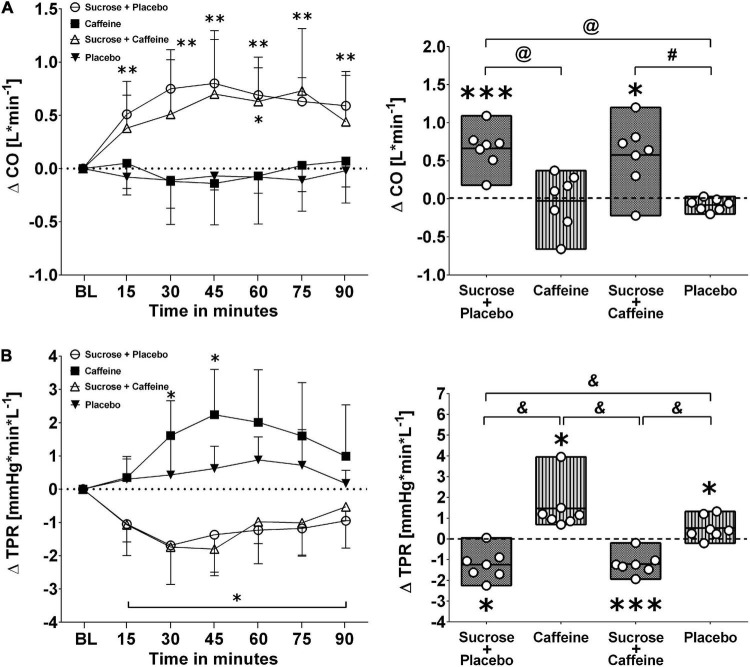
Left panels **(A,B)**: Time course for changes in cardiac output (CO) and total peripheral resistance (TPR) in response to the four drinks. Each mean value (i.e., 15-min blocks) corresponds to the average over the respective interval that was subtracted by its baseline (BL) values. Data are presented as means ± standard deviation. Right panels **(A,B)**: Boxes (minimum to maximum values) represent individual mean responses (open circles) averaged over 90 min post-drink and subtracted by baseline values for each drink, respectively. In both panels, asterisks show statistically significant differences over time from baseline values (both panels) at *p < 0.05, **p < 0.01, and ***p < 0.005. For right panels only, ^#^*p* < 0.05, ^@^*p* < 0.01, and *^&^p* < 0.005 represent statistically significant differences between responses to the drinks as analyzed by one-way repeated measures analysis of variance with Holm–Sidak’s *post hoc* testing.

**FIGURE 4:**
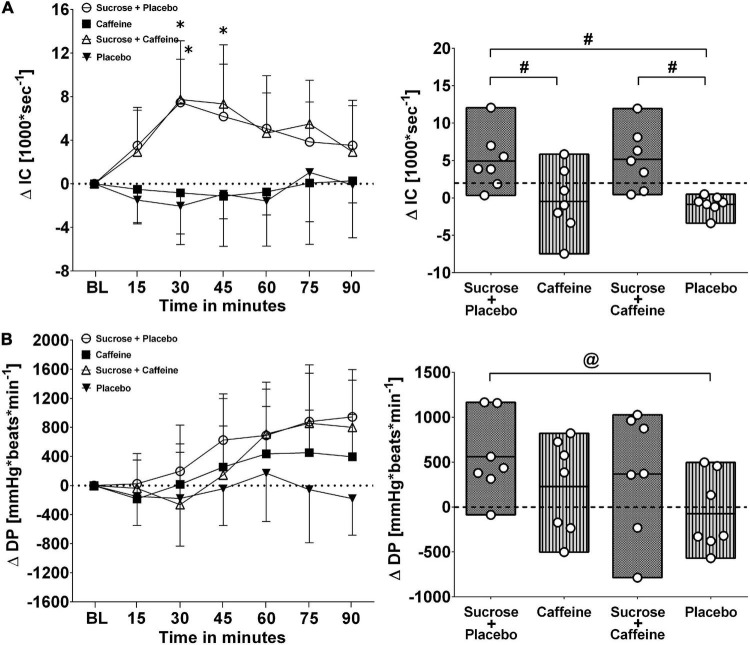
Left panels **(A,B)**: Time course for changes in index of contractility (IC) and double product (DP) in response to the four drinks. Each mean value (i.e., 15-min blocks) corresponds to the average over the respective interval that was subtracted by its baseline (BL) values. Data are presented as means ± standard deviation. Right panels **(A,B)**: Boxes (minimum to maximum values) represent individual mean responses (open circles) averaged over 90 min post-drink and subtracted by baseline values for each drink, respectively. In both panels, asterisks show statistically significant differences over time from baseline values (both panels) at *p < 0.05. For right panels only, **^#^***p* < 0.05 and **^@^***p* < 0.01 represent statistically significant differences between responses to the drinks as analyzed by one-way repeated measures analysis of variance with Holm–Sidak’s *post hoc* testing.

Figures 3, 4 depict changes in cardiac output and total peripheral resistance as well as changes in index of contractility and double product in response to the ingestion of four test drinks. Two-way repeated measures ANOVA analysis provided evidence for a significant treatment versus time interaction effect in averaged responses to cardiac output (*p* < 0.005), total peripheral resistance (*p* < 0.005), index of contractility (*p* < 0.005), and double product (*p* = 0.007).

Ingestion of sugar-sweetened drinks with and without caffeine decreased total peripheral resistance similarly over time with nadir responses between min 30 and 45 and a gradual return toward baseline values thereafter, while caffeine-only showed opposing resistance responses that peaked similarly around min 30–45 (Figure 3, panel B left). Indeed, responses were significantly higher with caffeine-only compared to sucrose + caffeine-placebo (*p* < 0.01) as well as to sucrose + caffeine (*p* < 0.01) (Figure 3, panel B right). Additionally, sugar-sweetened drinks with and without caffeine, but not caffeine-only, increased significantly cardiac output over time and throughout the study duration (*p* = 0.006, respectively) (Figure 3, panel A left). Cardiac output responses were significantly higher with sucrose + caffeine-placebo (*p* < 0.005) and sucrose + caffeine (*p* = 0.04) compared to the caffeine placebo control (Figure 3, panel A right). Similar time responses were observed for index of contractility where sucrose + caffeine and sucrose + caffeine-placebo significantly increased its values (Figure 4, panel A left). Contractility responses were significantly higher for sucrose + caffeine-placebo compared to caffeine-only (*p* = 0.03) and showed a trend for higher sucrose + caffeine responses (*p* = 0.06) (Figure 4, panel A right). The sucrose drink tended to increase double product over time (Figure 4, panel B left).

## Discussion

This study explored cardiovascular changes in response to a sucrose-sweetened and caffeinated beverage at dosages similar to commercially available products. In order to execute this, a caffeine-placebo controlled, randomized crossover test design was chosen to investigate cardiovascular responses to four different test drinks [each treatment contained 500 mL water plus either (*A*) sucrose 60 g plus caffeine-placebo capsule, (*B*) caffeine capsule 50 mg, (*C*) sucrose 60 g plus caffeine capsule 50 mg, or (*D*) placebo capsule] in young and non-obese men. The results of the present study demonstrated caffeine-induced vascular effects (with increases in vascular resistance and blood pressure) that were dampened in the presence of sucrose. However, cardiac effects (i.e., changes in heart rate, stroke volume, cardiac output, and index of contractility) increased to a similar magnitude for sucrose + caffeine-placebo and sucrose + caffeine test drinks and tended to be lower in response to the caffeine-only drink. Taken together, these results suggest that the blood pressure-elevating effect of a low-dose caffeinated beverage is dampened in the presence of sucrose, which is possibly due to its peripheral vasodilatory properties. However, our observed sucrose-induced vascular effect is at the expense of a tendency to increased cardiac work.

The consumption of sugar-sweetened and caffeinated beverages still attracts millions of people all around the world. This clear trend is demonstrated by rising sale numbers of worldwide soft drink (18) and energy drink sales (18) as well as by predicted growth rates for the global energy drink market segment until 2026 (19). Consumption of sugar-sweetened beverages is associated with negative metabolic and cardiovascular profiles and increased risk of overweight/obesity (20–24), type 2 diabetes (4, 21), and cardiovascular diseases (3). Moreover, a recent study investigated the influence of caffeine on the consumption of sugar-sweetened beverages and observed that the addition of a moderate-dose caffeine increased the consumption of the sugar-sweetened beverage (25). Although cross-sectional and epidemiological studies suggest such a link, controlled human studies for the hemodynamic response to sugar-sweetened and caffeinated beverages are still scarce. Our laboratory recently provided evidence that each type of sugar found in typical sugar-sweetened beverages, i.e., glucose (8, 9, 26, 27), fructose (8, 26), sucrose (8), and galactose (28), elicits distinct short-term vascular and cardiac effects in young men. In this context, sucrose, which is comprised of glucose and fructose at equimolar amounts, produced hemodynamic actions that were more related to glucose rather than fructose (8). In extension to the aforementioned findings, this study provides evidence that caffeine-induced elevations in blood pressure can be dampened by the presence of sucrose at amounts similar (10, 11) to commercially available soft drinks. One potential explanation for this observation is a glucose-induced rise of insulin blood levels, which is known to decrease systemic vascular resistance in a dose-dependent manner (29). Therefore, it is possible that the glucose moiety contained in the sucrose drinks increased insulin levels, which would in turn explain the observed blood pressure dampening effect due to the resistance-lowering effects of insulin. However, it is currently not known whether this contention is still valid in obese people and/or subjects with a perturbation in the glucose metabolism (i.e., impaired glucose tolerance or type 2 diabetes). Our laboratory recently provided evidence that young men with overweight/obesity and mild alterations in glucose metabolism, but not impaired glucose tolerance, showed attenuated peripheral vasodilation and diminished cardiac responses to a 75 g glucose drink (27). Therefore, future studies are highly encouraged to investigate the potential interaction effects of sugar with caffeine in overweight/obese populations with and without impaired glucose tolerance or type 2 diabetes.

Our previous studies have brought evidence that a commercially available sugar-sweetened energy drink (Red Bull) exerts decreases in vascular resistance together with significant blood pressure elevations (7, 30). Although our later study did not include a sugar + caffeine-placebo test, it was hypothesized that part of the observed blood pressure elevating effects observed could be due to an interaction effect between sugar and caffeine (7). However, in the current study, blood pressure parameters increased only in response to caffeine, but not to the sucrose + caffeine-placebo and sucrose + caffeine drinks. While blood glucose and insulin were not measured in the present study (a limitation to the study) it can be speculated that the dose of caffeine in our study (∼0.64 mg caffeine per kg body weight) was too low to substantially perturb glucose metabolism in response to the addition of 60 g of sucrose. Beaudoin and colleagues investigated caffeine-induced changes of glucose metabolism by using an oral glucose tolerance test, with caffeine concentrations starting at 1 mg per kg body weight, and observed a dose-dependent deterioration of whole body glucose disposal (31). Hence, it is possible that a higher caffeine dose, such as used by Miles-Chan and colleagues (7) (∼1.6 mg caffeine per kg body weight), could induce impaired removal of blood glucose with concomitant detrimental hemodynamic changes. Our findings warrant further investigation in the interaction of orally ingested caffeine and sugar in healthy subjects as well as in patients suffering from type 2 diabetes and/or cardiovascular diseases in order to facilitate the development of personalized and individual primary and secondary preventive dietary recommendations.

In addition to the aforementioned vascular effects of caffeinated and sugar-sweetened beverages, there is compelling evidence that the ingestion of liquid sugars with (6, 7) and without (8, 26–28) caffeine strongly affects cardiac processes. Our laboratory observed that a glucose/sucrose sweetened, but not an artificially sweetened energy drink, increased significantly heart rate, stroke volume, index of contractility [marker for myocardial contraction force (15)], and double product [marker for myocardial oxygen demand (17)] (7). In agreement with these findings, we observed similar time course changes in response to the sucrose + caffeine test drink for heart rate, stroke volume, cardiac output, index of contractility, and double product. However, peak responses were markedly higher in the study by Miles-Chan and colleagues (7) for heart rate (+ 87%) and double product (+ 61%), while peak responses for cardiac output (+ 26%) and stroke volume (+ 1%) were similar. Since the present study used markedly lower caffeine concentrations (∼0.64 versus ∼1.6 mg per kg body weight), but higher sugar concentrations (∼0.76 versus ∼0.52 g per kg body weight), it is possible that the larger caffeine dose (i.e., + 160%) by Miles-Chan and colleagues (7) contributed to the observed differences for peak heart rate and double product responses. In line with this contention stands the observation that orally ingested caffeine dose-dependently reduces whole body glucose disposal with consequentially increasing insulin levels (30), which could, at least in part, explain the observed higher myocardial oxygen demand in response to sugar-sweetened beverages with higher caffeine doses than the ones used in the present study. Interestingly, although caffeine acutely impairs insulin sensitivity (31), many observational studies have found that regular consumption of coffee was associated with a lower risk of type 2 diabetes (T2D). Since this was found in several studies for both caffeinated and decaffeinated coffee (32), this would suggest that the beneficial effect of regular coffee consumption on T2D could be related to other chemical components found in coffee or to other factors.

In conclusion, this study investigated in young/non-obese men potential interaction effects of caffeine and sucrose at dosages similar to commercially available soft drinks. We provide evidence that the blood pressure-elevating effect of 50 mg of caffeine is dampened in the presence of 60 g sucrose possibly through reductions in vascular resistance. However, increases in resting heart rate, cardiac output, and a marker for cardiac contractility tended to be higher in response to the sucrose drinks with little effect of adding caffeine, thereby indicating an adverse cardiac profile in response to sucrose-sweetened caffeinated beverages. Therefore, the caffeine and sucrose moiety investigated here in young and non-obese men exerts distinct hemodynamic effects with no apparent potentiation.

## Scope and study limitations

This study, albeit using a small sample size of seven non-obese healthy men, clearly shows an increase in blood pressure following caffeine ingestion while the addition of sucrose directly diminishes these effects potentially through peripheral vasodilatory effects. This information is important and relevant as the consumption of sweetened caffeinated drinks continues to increase. Nevertheless, this study has certain limitations. As this study was planned as a pilot, only a small number of non-obese healthy men were included. In the future, adding women will be important as well as extending these experiments to other groups such as those that are affected by obesity and/or diabetes.

## Data availability statement

The raw data supporting the conclusions of this article will be made available by the authors, without undue reservation.

## Ethics statement

The studies involving human participants were reviewed and approved by Commission Cantonale D’Ethique, Canton de Vaud, Switzerland (ethics number 239/15). The patients/participants provided their written informed consent to participate in this study.

## Author contributions

EG and J-PM contributed to the conception and design of the study. YR and EG organized the data and performed data analysis. EG wrote the first draft of the manuscript. All authors contributed to the article and approved the submitted version.

## Footnotes

1
http://www.random.org/sequences/
